# Synthesis and Evaluation of a Molecularly Imprinted Polymer for 2,4-Dinitrophenol

**DOI:** 10.3390/ijms10010354

**Published:** 2009-01-22

**Authors:** Nor Dyana Zakaria, Nor Azah Yusof, Jelas Haron, Abdul Halim Abdullah

**Affiliations:** Chemistry Department, Faculty of Science, University Putra Malaysia, 43400, Serdang, Selangor, Malaysia. E-Mails: myaminx@gmail.com (N. Z.); halim@science.upm.edu.my (A. A.); mdjelas@science.upm.edu.my (J. H.)

**Keywords:** Molecular Imprinted Polymer, 2,4-dinitrophenol, separation

## Abstract

Molecular imprinted polymers (MIP) are considered one of the most promising selective and novel separation methods for removal phenolic compound in wastewater treatment. MIP are crosslinked polymeric materials that exhibit high binding capacity and selectivity towards a target molecule (template), purposely present during the synthesis process. In this work MIP were prepared in a bulk polymerization method in acetonitrile using 2,4-dinitrophenol, acrylamide, ethylene glycol dimethacrylate, and benzoyl peroxide as template, functional monomer, cross-linker and initiator, respectively. An adsorption process for removal of nitrophenol using the fabricated MIP was evaluated under various pH and time conditions. The parameters studied for 2,4-dinitrophenol includes adsorption kinetics, adsorption isotherm, and selectivity. The maximum adsorption of nitrophenol by the fabricated MIP was 3.50 mg/g. The adsorption of 2,4-dinitrophenol by the fabricated MIP was found effective at pH 6.0. A kinetics study showed that nitrophenol adsorption follows a second order adsorption rate and the adsorption isotherm data is explained well by the Langmuir model.

## 1. Introduction

The presence of phenol derivatives in food, in ground and surface waters poses a significant danger to health and the environment because of their inherent toxicity. They are considered as priority pollutants in the US EPA list and their discharge in the aquatic environment is becoming increasingly restricted. A phenol concentration of 1 mg/L or greater affects aquatic life and can cause major risks to human health. Therefore, in most cases stringent effluent discharge limit of less than 0.5 mg/L are imposed. In this sense, the US EPA has established a limit concentration of 1 μg/L for phenolic compounds for drinking water [1]. Methods that have been developed for the removal of nitrophenols from water include microbial degradation and chemical oxidation. Slow reaction rates, disposal of sludge, and control of temperature and pH are all drawbacks associated with microbial degradation [2] while chemical oxidation is only economically feasible at high concentrations [3]. Many methods are available for removal of phenolic compound, such as by an electro-assisted process employing a modified electrode [4–5], using chemical sorbents such as Amberlite^®^ XAD-4 [6], a hypercrosslinked polymeric compound [7–8] and biosorbents [9–10]. Among the many available methods for removal of phenolic compounds, MIP is one that offers excellent selectivity towards the target molecules. The good selectivity obtained with MIP-based separation materials has led to them being considered as promising selective sorbents for SPE. They are as a result now being extensively investigated as highly selective SPE sorbents for the washing and the preconcentration of samples prior to analysis [11].

MIPs are being exploited in an increasing number of applications that include their use as “tailor-made” separation materials, as antibody/receptor binding site mimics in recognition and assay systems, as enzyme mimics for catalytic applications, as recognition elements in sensors as well as in facilitated chemical synthesis [12–14]. To date, their most extensively investigated application has been as separation materials for the analysis of numerous compounds such as drugs [15–16], pesticides [17–18] and amino acids [19].

MIP is a technique for making selective template binding sites in synthetic polymers by using a molecular template. Target molecules (i.e. phenolics) can be used as templates for imprinting cross-linked polymers. After the removal of template, the remaining polymer is more selective for the target molecule(s) compared to a non-imprinted polymer. The selectivity of the polymer depends on various factors such as the size and shape of the cavity and rebinding interactions. Covalent interactions, non-covalent interactions such as hydrogen bonding, π–π bonding and hydrophobic interaction, electrostatic interactions and metal ion co-ordination can be exploited to organize the functional monomers around the template [20].

In this work, bulk polymerization was employed to prepare MIP for 2,4-dinitrophenol in acetonitrile using 2,4-dinitrophenol, acrylamide, ethylene glycol dimethacrylate (EGDMA), and benzoyl peroxide (BPO) as template, functional monomer, cross-linker, and initiator, respectively. The characteristics of the obtained polymers were analyzed using Fourier Transformed Infrared (FTIR) spectroscopy and a Nanophox particle size analyzer. The parameters studied include pH, kinetics and the adsorption process isotherms. The selectivity of the obtained particles was elucidated by studying the different rebinding capabilities of 2,4-dinitrophenol samples and structurally related compounds. The fabricated MIP can be used for separation, preconcentration and analysis of trace phenolic compound samples.

## 2. Results and Discussion

### 2.1. Characterization of MIP

**Figure 1.**
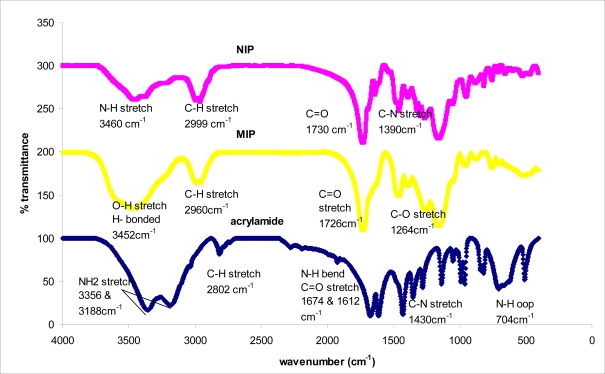
IR spectra for MIP, NIP and monomer.

**Figure 2.**
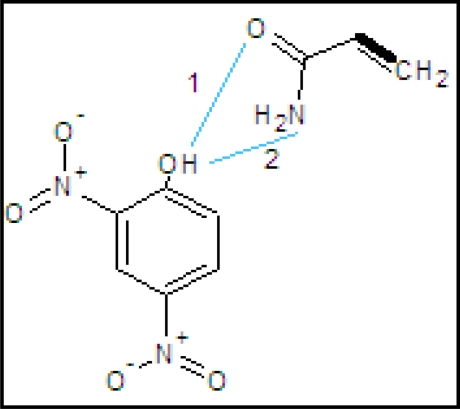
Proposed interaction between monomer (acrylamide)and the template (2,4-dinitrophenol).

**Figure 3.**
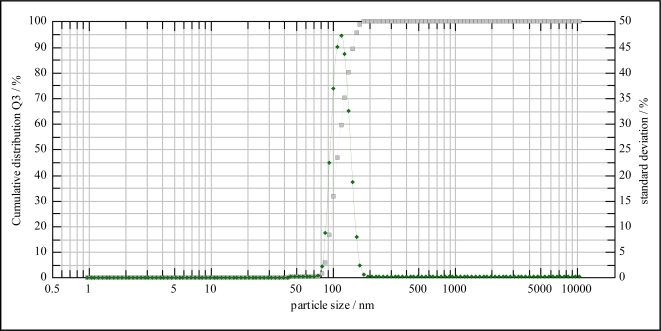
The MIP particles size using a Nanophox particle size analyzer.

The IR spectra of the 2,4-dinitrophenol imprinted polymer, non-imprinted polymer (NIP) and monomer materials were recorded using the KBr pellet method. The spectral differences between MIP and monomer acrylamide are shown in Figure 1. Both spectra indicated the presence of CO carbonyl groups. However, the spectrum of acrylamide in the 3,356–3,188 cm^−1^ range showed the characteristic NH_2_ stretch of the amide group. Fortunately, the interaction between template and monomer gave changeable peaks in the MIP spectrum, which showed a board OH stretching peak at 3,452 cm^−1^ and CH stretching at 2,960 cm^−1^, while the CH stretching in the spectrum of acrylamide was at 2,802 cm^−1^. For the NIP, the N-H stretching vibration occurs in the 3,460 cm^−1^ range. The C-N stretching absorption occurs in the 1390 cm^−1^ region. C=O stretching vibrations occur in the 1,730 and 1,726 cm^−1^ range, which shows a very strong ketone group band. The amide shows a very strong band for the C=O group that appears in the 1,674–1,612 cm^−1^ range. N-H bending bands, in solid primary amides give strong bending vibrational bands in the 1,674–1,612 cm^−1^ range. They often nearly overlap with the C=O stretching bands. Primary amides give other bending bands and a very broad band around 704 cm^−1^. A C-N stretching band appears at about 1,430 cm^−1^ for arylamide. The broadening at 3,400 cm^−1^ indicates that a hydrogen bonding interaction takes place between the phenolic hydroxyl group and the carbonyl from the amido group (as shown in Figure 2). The size of the MIP particles is in the range of 130.02 nm, as shown in Figure 3 (data from the particle size analyzer).

### 2.2. pH Study

**Figure 4.**
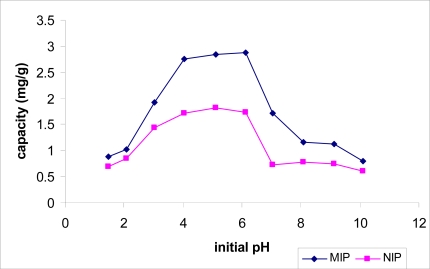
Effect of pH on adsorption of 2,4-dinitrophenol by MIP.

The pH of the solution is one of the most important variables affecting the adsorption process. Adsorption of 2,4-dinitrophenol was measured at different pH values (1–10). At every pH range, the 2,4-dinitrophenol capacity on MIP was higher than on NIP. Figure 4 shows that the maximum adsorption of 2,4-dinitrophenol was found to be 2.88 mg/g under acidic conditions at pH 6.0. The capacity decreased when the pH values increased.

### 2.3. Kinetic Study

**Figure 5.**
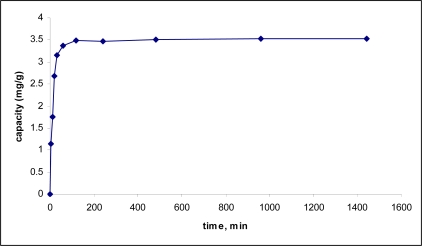
Adsorption rate of 2,4-dinitrophenol by MIP.

The rate of the adsorption of 2,4-dinitrophenol by the MIP was measured as a function of time. The result of this kinetics study is shown in Figure 5. The 2,4-dinitrophenol sorption process was rapid in the initial stage and rather slow when approaching equilibrium. The maximum adsorption occurred after 1 hour at a capacity around 3.50 mg/g.

The sorption kinetic data of 2,4-dinitrophenol was analyzed using the Langergen first order rate model. The equations involved are as below:

(1) $$\frac{dq_{t}}{d_{\text{t}}}=k_{1}(q_{e}-q)$$

where k_1_ (min^−1^) is the rate constant of pseudo-first-order sorption, q_t_ denotes the amount of 2,4-dinitrophenol sorption (mg/g) at time, t (min) and q_e_ denotes the amount of 2,4-dinitrophenol adsorption (mg/g) at equilibrium. After definite integration and applying the initial conditions q_t_ = 0 at t = 0 and q_t_ = q_t_ at t = t, Equation (1) becomes:

(2) $$\ln(q_{e}-q_{t})=\ln(q_{e})-k_{1}t$$

In addition, a pseudo-second-order equation based on adsorption equilibrium capacity may be expressed in the form:

(3) $$\frac{dq_{t}}{dt}=k_{2}(q_{e}-q_{t}{)}^{2}$$

where k_2_ is the rate constant of pseudo-second-order sorption. Integrating Equation 3 and applying the initial conditions,

(4) $$\frac{1}{\left(q_{e}-q_{t}\right)}=\frac{1}{q_{e}}+k_{2}t$$

or equivalently:

(5) $$\frac{t}{q_{t}}=\frac{1}{{k_{2}q_{e}}^{2}}+\frac{t}{q_{e}}$$

The plot t/q versus t should give a straight line if second order kinetics are applicable and the values q_e_ and k_2_ can be calculated from the slope and the intercept of the plot, respectively.

**Figure 6.**
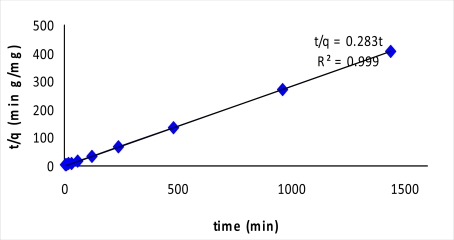
Second order kinetic plot for adsorption of 2,4-dinitrophenol by MIP.

The equilibrium removal of 2,4-dinitrophenol was mathematically expressed in terms of the adsorption kinetics. The data does not fall on a straight line and had a low correlation coefficient, indicating the first order kinetic model is less appropriate. On the other hand, the plot of t/q against t results in a high correlation coefficient (0.999) (Figure 6). This indicates that 2,4-dinitrophenol adsorption by using MIP follows a second order kinetics reaction.

### 2.4. Adsorption Isotherm Study

**Figure 8.**
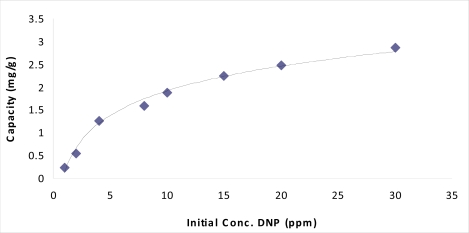
Adsorption capacity of various concentration of 2,4-dinitrophenol by MIP.

The amount of materials adsorbed is determined as a function of the concentration at constant temperature that could be explained in adsorption isotherms. Adsorption isotherm was measured for 2,4-dinitrophenol with MIP using the optimized conditions and the results are shown in Figure 8. The maximum adsorption capacity was observed to be 2.87 mg/g. Equations that are often used to describe the experimental isotherm data were developed by Langmuir and Freundlich. These equations are commonly used for describing sorption equilibrium for water and wastewater treatment applications.

The Langmuir model is probably the best known and most widely applied adsorption isotherm. It has produced good agreement with a wide variety of experiment data and may be represented as follows:

(6) $$q_{e}=\frac{q_{m}bC_{e}}{1+bC_{e}}$$

The above equation can be rearranged to the following linear form:

(7) $$\frac{C_{e}}{q_{e}}=\frac{C_{e}}{q_{m}}+\frac{1}{bq_{m}}$$

where C_e_ is the equilibrium concentration (mg/L), q_e_ the amount of 2,4-dinitrophenol adsorbed at equilibrium (mg/g), q_m_ is amount of 2,4-dinitrophenol sorbed for a complete monolayer (mg/g), b is a constant related to the energy or net enthalpy of sorption (L/mg). The adsorption data were analyzed using the linear form, Equation (7) of the Langmuir isotherm. The plots of specific sorption, C_e_/q_e_, against the equilibrium concentration, C_e_, for MIP are shown in Figure 8.

The Freundlich expression is an exponential equation and therefore assumes that as the sorbate concentration increases, the concentration of sorbate on the adsorbent surface will increase also:

(8) $$q_{e}={K_{f}C_{e}}^{1/n}$$

The equation is frequently used in the linear form by taking the logarithm of both sides as:

(9) $$\text{log}\;q_{e}\;=\;\text{log}\;K_{f}\;+\;1/n\;\text{log}\;C_{e}$$

where K_f_ and n are isotherm constant, respectively.

**Figure 9.**
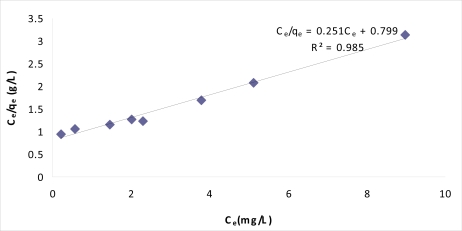
Langmuir plot for adsorption of 2,4-dinitrophenol by MIP.

The applicability of the Freundlich sorption is also analyzed by plotting log q_e_ versus log C_e_. To determine the constant K_f_ and n, the linear form of equation, Eq (10) can be used. Compared to Freudlich, the Langmuir plots have a higher correlation coefficient of 0.985. It can be confirmed that 2,4-dinitrophenol adsorption by MIP follow the Langmuir Model with a maximum adsorption capacity of 2.87 mg/g at room temperature (Figure 9).

### 2.5. Selectivity Study

Competitive adsorptions of phenol/2,4-dinitrophenol, 3-chlorophenol/2,4-dinitrophenol and 2,4-dichlorophenol/2,4-dinitrophenol from their mixtures were conducted using imprinted and non-imprinted polymer. The effect of imprinting on selectivity was defined as:

(10) $$K_{d}=\frac{[C_{i}-C_{f}]\times \nu }{M}$$

where K_d_ is distribution coefficient, Ci and Cf the initial and final solution concentration, respectively. v (mL) is the volume of the solution and M (g) is the weight of MIP.

The selectivity coefficient for the binding of phenol compound in the presence of competitor species can be obtained from equilibrium binding data according to:

(11) $$K_{d}\;=\;\frac{K_{d}(2,4-dinitrophenol)}{K_{d}(phenolic)}$$

where k is the selectivity coefficient and (phenolic) represents the compounds phenol, 3-chlorophenol and 2,4-dichlorophenol. A comparison of k values of the imprinted polymers with those phenolic compounds allows an estimate of the effect of imprinting on selectivity. A relative selectivity coefficient *k’* can be defined as:

(12) $$k\prime =\frac{kimpr\;\mathrm{int}\;ed}{kcontrol}$$

**Figure 11.**
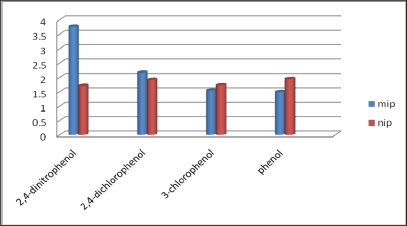
Selectivity study of 2,4-dinitrophenol by MIP in the presence of various phenolic compound.

The binding capacity adsorption of MIP for 2,4-dinitrophenol is higher than NIP. Table 1 summarizes K_d_, k and *k’* values of phenol, 3-chlorophenol and 2,4-dichlorophenol with respect to 2,4-dinitrophenol. The k’ values for MIP of phenol/2,4-dinitrophenol, 3-chlorophenol/2,4-dinitrophenol and 2,4-dichlorophenol/2,4-dinitrophenol were 2.88, 2.46 and 1.95, respectively, indicating a good selectivity towards 2,4-dinitrophenol. The selectivity of the MIP and NIP towards 2,4-dinitrophenol in the presence of phenol, 3-chlorophenol and 2,4-dichlorophenol were also evaluated and the results (Figure 11) showed that the MIP has a greater selectivity towards the target 2,4-dinitrophenol compared to NIP.

## 3. Experimental Section

### 3.1. Materials

Acrylamide, 2,4-dinitrophenol, ethylene glycol dimethacrylate, and benzoyl peroxide were obtained from Fluka (Switzerland). All other chemicals were of reagent grade and purchased from Merck (Germany).

### 3.2. Preparation of 2,4-dinitrophenol-imprinted Polymer

Molecular imprinted polymer was prepared using the non-covalent approach. The template, 2,4-dinitrophenol (1.0 mmol) was dissolved in acetonitrile (30 mL) in a flask. The functional monomer acrylamide (5.0 mmol), the cross-linker ethylene glycol dimethacrylate (EDGMA) (15 mmol) and the initiator benzoyl peroxide (BPO, 1.6 mmol) were then added to the flask. After degassing and nitrogen purging for 10 minutes, the flask was sealed and the contents allowed to polymerize in a water bath at 70 °C for 24 hours. The obtained bulk polymers were crushed and ground to obtain regularly sized particles between 80–100 μm. The template molecules were leached out using methanol-acetic acid (1%) with a 2:1 ratio three times. The particles were then extensively washed with water until no more 2,4-dinitrophenol was released. Non-imprinted polymer (NIP) was prepared and treated with the same method, but in the absence of 2,4-dinitrophenol.

### 3.3. Adsorption Studies

Adsorption of 2,4-dinitrophenol from aqueous solutions was investigated in batch experiments. Effects of pH, adsorption isotherm, kinetics and selectivity of the fabricated MIP were examined. MIP (0.05 g) was shaken for 1 hour in 10 ppm 2,4-dinitrophenol (20 mL). The pH was adjusted with hydrochloric acid (HCl) or sodium hydroxide (NaOH). The concentration of 2,4-dinitrophenol in the aqueous after desired treatment periods was analyzed using UV-Vis Spectroscopy. The adsorption capacity was calculated as below:

(13) $$q=\frac{(C_{o}-C_{e})\times V}{M}$$

where q (mg/g) is the amount of total adsorption of 2,4-dinitrophenol, C_o_ and C_e_ are initial and equilibrium concentration of 2,4-dinitrophenol in solution (mg/L), respectively. V (L) is the volume of the solution and M (g) is the weight of MIP.

The adsorption kinetics were studied by stirring MIP (0.05 g) in 10 ppm 2,4-dinitrophenol (20 mL) for different times (5, 10, 20, 30, 60, 120, 240, 480, 960 and 1440 min). The adsorption isotherms were studied by stirring MIP (0.05 g) in 2,4-dinitrophenol solution (20 mL) at different concentration (1, 2, 4, 8, 10, 15, 20 and 30 mg/L).

Selectivity of the fabricated MIP towards phenol, 3-chlorophenol and 2,4-dichlorophenol with respect to 2,4-nitrophenol was studied. A solution (25 mL) containing 10 mg/L (for each compound) was mixed together and treated with MIP (0.05 g) at room temperature. The concentration of the phenolic compounds after treatment was measured by UV-Vis Spectroscopy. The binding capacity and the distribution coefficient were calculated.

### 3.4. Characterization of MIP

Characterizations of the imprinted polymer were carried out using Fourier Transformed Infrared (FTIR) spectroscopy. The imprinted polymer particles (~0.01g) were thoroughly mixed with KBr and pressed into a pellet and the FTIR spectrum was recorded. A Nanophox particle size analyzer was used to determine the size of the particles. The MIP (~0.01g) in deionized water (10 mL) was sonicated for 10 minutes before being analyzed with the Nanophox particle size analyzer.

## 4. Conclusions

The potential use of a molecular imprinted polymer prepared from acrylamide as functional monomer for the effective removal of 2,4-dinitrophenol from aqueous solutions was investigated. Adsorption of 2,4-dinitrophenol by MIP-acrylamide was found to be effective at pH 6.0. In the kinetic study, it was found that the rate of adsorption 2,4-dinitrophenol increased rapidly in the initial stage and then reduced until it reached equilibrium. Compared a to pseudo first order model, a second order kinetic equation gave a better fit for the adsorption kinetic data. The adsorption increased with increasing initial concentration. The results showed that the adsorption process obeys a Langmuir adsorption isotherm.

The selectivity experiments showed that the MIP is selective towards 2,4-dinitrophenol in the presence of phenol, 3-chlorophenol and 2,4-dichlorophenol interferences. The fabricated MIP has potential use in a preconcentration process in trace analysis of 2,4-dinitrophenol and also in development of columns for chromatograph. Furthermore MIP is inexpensive and easy to prepare.

## Figures and Tables

**Table 1.** Parameters involved in selectivity study of the MIP and NIP towards different kind of phenolic compound.

	K_d (MIP)_ (mg/g)	K_d (NIP)_ (mg/g)	k (MIP)	k_(NIP)_	*k’*
2,4-Dinitrophenol	3.8	1.7			
Phenol	1.5	1.9	2.5	0.88	2.88
3-Chlorophenol	1.5	1.7	2.4	0.99	2.46
2,4-Dichlorophenol	2.2	1.9	1.7	0.89	1.95

## References

[1] Puig D, Barcelo D (1997). Determination of polar priority phenols at parts per trillion levels in water using on-line liquid-solid extraction followed by liquid chromatography with coulimetric detection. J. Chromatogr. A.

[2] Tomei MC, Rossetti S, Annesini MC (2006). Microbial and kinetic characterization of pure and mixed cultures aerobically degarding 4-nitrophenol. Chemosphere.

[3] Wu Z, Cong Y, Zhou M, Ye Q, Tan T (2002). Removal of phenolic compounds by electroassisted advanced process for wastewater purification. Kor J. Chem. Eng.

[4] Zaggout FR, Ghalwa NA (2008). Removal of *o*-nitrophenol from water by electrochemical degradation using a lead oxide/titanium modified electrode. J. Environ. Manage.

[5] Belaid C, Kallel M, Khadhraou M, Lalleve G, Elleuch B, Fauvarque JF (2006). Electrochemical treatment of olive mill wastewaters: Removal of phenolic compounds and decolourization. J. Appl. Electrochem.

[6] Ku Y, Lee KC (2007). Removal of phenols from aqueous solution by XAD-4 resin. J. Hazard. Mater.

[7] Oh CG, Ahn JH, Ihm SK (2004). Adsorptive removal of phenolic compounds by using hypercrosslinked polystyrenic beads with bimodal pore size distribution. Sep. Purif. Technol.

[8] El-Hamshary H, El-Sigeny S, Manal F, Taleb A, El-Kelesh NA (2007). Removal of phenolic compounds using (2-hydroxyethylmethacrylate/acrylamidopyridine) hydrogel prepared by gamma radiation. Sep. Purif. Technol.

[9] Kadhim H, Graham C, Barrat P, Evans CS, Rastall RA (1999). Removal of phenolic compounds in water using Coriolus versicolor grown on wheat bran. Enzyme Microb. Technol.

[10] Damaris NM, Shiundu PM, Ndonye RM, Kamau GN (2002). Adsorption and detection of some phenolic compounds by rice husk ash of Kenyan origin. Environ. Monit.

[11] Caro E, Marcé RM, Cormack PG, Sherrington DC, Borull F (2003). On-line solid-phase extraction with molecularly imprinted polymers to selectively extract substituted 4-chlorophenols and 4-nitrophenol from water. J. Chromatogr. A.

[12] Ricardo C, Tarley T, Kubota LT (2005). Molecularly-imprinted solid phase extraction of catechol from aqueous effluents for its selective determination by differential pulse voltammetry. Anal. Chim. Acta..

[13] Guney O, Yilmaz Y, Pekcan O (2002). Metal ion templated chemosensor for metal ions based on flouresence quenching. Sens. Actuat. B.

[14] Kataky R, Morgan E (2003). Potential of enzyme mimics in biomimetic sensors: A modified cyclodextrin as a dehydrogenase enzyme mimic. Biosens. Bioelectron.

[15] Weiss R, Molinelli A, Jakusch M, Mizaikoff B (2001). Molecular imprinting and solid phase extraction of flavonoid compounds. Bioseparation.

[16] Walshe M, Garcia E, Howarth JM, Smyth R, Kelly MT (1997). Separation of the enantiomers of propranolol by incorporation of molecularly imprinted polymer particles as chiral selectors in capillary electrophoresis. Anal. Commun.

[17] Yang G, Liu H, Wang M, Liu S, Chen Y (2006). Chromatographic characterization and solid-phase extraction on diniconazole-imprinted polymers stationary phase. React. Funct. Polym.

[18] Zhang H, Song T, Zong F, Chen T, Pan C (2008). Synthesis and characterization of molecularly imprinted polymers for phenoxyacetic acids. Int. J. Mol. Sci.

[19] Shim YH, Yilmaz E, Lavielle S, Haupt K (2004). Chiral recognition and separation of β^2^-amino acids using non-covalently molecularly imprinted polymers. Analyst.

[20] Adil ED, Sener I (2004). Removal of phenolic compound with nitrophenol-imprinted polymer based on π-π and hydrogen-bonding interactions. Sep. Purif. Technol.

